# Loss of* LLGL1* Expression Correlates with Diffuse Gastric Cancer and Distant Peritoneal Metastases

**DOI:** 10.1155/2019/2920493

**Published:** 2019-04-01

**Authors:** Alexander Desuki, Frank Staib, Ines Gockel, Markus Moehler, Hauke Lang, Stefan Biesterfeld, Annett Maderer, Peter R. Galle, Martin R. Berger, Carl C. Schimanski

**Affiliations:** ^1^First Department of Internal Medicine, Johannes Gutenberg University, Langenbeckstraße 1, 55131 Mainz, Germany; ^2^Third Department of Internal Medicine, Johannes Gutenberg University, 55131 Mainz, Germany; ^3^Department of Internal Medicine, Marienhospital Darmstadt gGmbH, Martinspfad 72, 64285 Darmstadt, Germany; ^4^Department of Visceral, Transplantation, Thoracic and Vascular Surgery, University of Leipzig, Liebigstraße 20, 04103 Leipzig, Germany; ^5^Department of Abdominal and General Surgery, Johannes Gutenberg University, Langenbeckstraße 1, 55131 Mainz, Germany; ^6^Institute of Pathology, Johannes Gutenberg University, Langenbeckstraße 1, 55131 Mainz, Germany; ^7^Toxicology and Chemotherapy Unit, German Cancer Research Center, Im Neuenheimer Feld 280, 69120 Heidelberg, Germany; ^8^Second Department of Internal Medicine, Klinikum Darmstadt GmbH, Grafenstraße 9, 64283 Darmstadt, Germany

## Abstract

**Background:**

Loss of* LLGL1 *has been associated with loss of cellular adhesion and dissemination of cells from colorectal cancer and malignant melanoma. Regulation and relevance of* LLGL1* were analyzed in gastric cancer patients with lymphatic and distant dissemination. Furthermore,* LLGL1 *expression was analyzed in relation to the cellular adhesion protein* E-cadherin*.

**Methods:**

* LLGL1* and* E-cadherin* transcription levels were evaluated in 56 gastric cancer patients and five gastric cancer cell lines. IHC staining for* LLGL1* was performed on 39 gastric cancer specimens.* LLGL1* was stably transfected into* LLGL1* negative gastric cancer cell line SNU16 (del(17) (p11.2)) for functional* in vitro* assays and a xenograft bioassay.

**Results:**

Gastric cancer specimens and cell lines displayed* LLGL1* and* E-cadherin* expression levels with variable intensity. In gastric mucosa,* LLGL1* exhibited weak cytoplasmic and strong cortical staining. Loss of* LLGL1* expression occurred in 65% of gastric cancers and significantly correlated with loss of* E-cadherin* expression (P=0.00009). Loss of* LLGL1* expression was associated with the diffuse type of gastric cancer (P=0.029) with peritoneal carcinomatosis (M1; P=0.006) and with female gender (P=0.017). Stable reexpression of* LLGL1* in SNU16 cells significantly increased both plastic surface adhesion and extracellular matrix proteins laminin and fibronectin, but had no impact on* in vitro* proliferation, apoptosis, or invasion or on* in vivo* proliferation or differentiation in our xenograft bioassay.

**Conclusion:**

* LLGL1* is coexpressed with* E-cadherin.* Loss of expression of either protein is associated with diffuse gastric cancer and peritoneal metastases.* LLGL1* does not impact on proliferation or epithelial-mesenchymal transition (EMT) rather increasing cellular adhesion.

## 1. Introduction

Gastric cancer incidence has decreased steadily in industrialized countries over the last years. However, gastric cancer still ranks among the most common causes of cancer and its mortality rate remains high [1–3]. The current gold standard therapy with curative intention is radical surgical resection with standardized D2-lymphadenectomy. Despite considerable improvements achieving R0 resections patients still require (neo)adjuvant chemotherapeutic strategies as they are still at high risk for local recurrences and early lymph node or systemic metastases [4, 5].

Accepted risk factors for gastric cancer are chronic atrophic gastritis, chronic H. pylori infection, and hypertrophic gastropathy among others [6]. Molecular determinants occurring during the development of gastric cancer include mutations of tumor suppressor genes (*E-cadherin, APC*,* DCC*,* Rb*,* p53*), oncogenes (*K-ras*), and mismatch repair genes (*MLH-1*) [7–10].

Tumor dissemination results from loss of cellular adhesion, chemotaxis, and neoangiogenesis. Junctions between epithelial cells have* communicating functions* such as gap junctions, are* anchoring junctions* such as desmosomes and adherens junctions, or are* sealing junctions* such as zonula occludens or tight junctions. Adherens junctions segregate the apical from the basolateral membrane domains. The predominant protein of adherens junctions is* E-cadherin*, a transmembrane protein stabilizing the basolateral cell-cell contact. Loss of* E-cadherin* expression has been linked to dissemination of various gastrointestinal malignancies [11, 12]. As early as in 1994, loss of* E-cadherin* expression was correlated with diffuse type gastric cancer [13]. Since then, multiple reports have described the association between diffuse gastric cancer and metastatic disease and also linked the loss of E*-cadherin* expression with familial gastric cancer [14, 15]. Loss of* E-cadherin* decreases cellular adhesion, resulting in a critical increase in cellular motility and migration [16].

Another relevant protein for cellular adhesion along the basolateral membrane domain is lethal giant larvae (*l(2)gl*) [17]. In* Drosophila* loss of* l(2)gl* results in loss of epithelial structure, malignant transformation of the brain hemispheres, and the imaginal discs and in growth of tumor masses resembling human cancers [18]. These tumors proliferate and migrate to distant sites upon transplantation into wild type* Drosophila,* thus acting like human metastatic cancers [19, 20]. Homologues of* l(2)gl* have been identified in diverse species such as rat, insect, worm, and man [21–23]. Remarkably, the particular function of* l(2)gl* is conserved among species, as shown by rescue of the* l(2)gl* mutation in Drosophila with the human homologue* LLGL1* [22, 24].

Evidence has also been published that mammalian* l(2)gl* regulates epithelial cell polarity and migration as a member of the polarity complex consisting of* Par6/Par3/atypical PKC* and* l(2)gl* [25–27].

In humans, highly related homologues of* l(2)gl, LLGL1,* and* LLGL2* have been identified, mapping to the short and long arm of chromosome 17.* LLGL1* has been located in a critical pericentromeric region, 17p11.2-12 containing cancer susceptibility genes for primitive neuroectodermal tumors [21]. Furthermore,* LLGL1* maps within the 17p interstitial deletion detected in mentally retarded children with Smith-Magenis syndrome [28, 29].

In 2005, loss of* LLGL1 *was associated with tumor-suppressive functions and was then linked with metastatic colorectal cancer, melanoma, endometrial cancer, hepatocellular cancer, pancreatic cancer, glioma, and lung cancer [30–36]. Overexpression of* LLGL1 in vitro *inhibited migration, increased cellular adhesion, lowered proliferation, and increased apoptosis [32, 37]. In addition,* LLGL1* could rescue its mutated respective* Drosophila* homologue, demonstrating a conserved tumor suppressor function [24]. Regarding* LLGL2, *reduced expression has been described in specimens of high grade pancreatic intraepithelial neoplasia, high grade gastric dysplasia, and carcinoma [37–40]. Interestingly, reduced basolateral* LLGL2* expression was associated with diffuse type gastric cancer and reduced E-cadherin expression [38, 41]. Taken together with the data presented in this paper, evidence is accumulating that both human homologues of Drosophila* l(2)gl* are involved in common human pathways, the inactivation of which promotes cancer dissemination.

The present study was performed to evaluate the role of* LLGL1* in human gastric carcinogenesis and to analyze the association and shared regulation with* E-cadherin* expression. We screened the transcription profile of* LLGL1* and* E-cadherin* in 5 human gastric cancer cell lines and 56 gastric carcinomas and performed additional IHC staining of 5 gastric mucosal samples and 39 gastric cancers. Functional* in vitro* assays with a stably* LLGL1* transfected cell line were performed to characterize the biological features of* LLGL1*. We then used the cell lines to induce subcutaneous xenograft tumors and assessed size and grading with respect to* LLGL1* expression.

## 2. Material and Methods

### 2.1. Cell Culture

For functional analyses, we studied the human gastric cancer cell lines AGS, NCI-N87, OE33, MKN45, and SNU16. All cell lines were cultured in DMEM supplemented with 10% FCS.

### 2.2. Tissue Source and Storage

Following ethics committee approval and signed informed consent, samples from the center of the tumor were obtained from 56 patients undergoing elective surgery for gastric cancer at the Department of Abdominal- and General Surgery, Johannes Gutenberg University, Mainz, Germany. All tissues were stored in cryovials, shock frozen in liquid nitrogen immediately after extirpation and stored at -80°C until further processing.

### 2.3. RNA Isolation and RT-PCR

RNA isolation was performed using the Qiagen RNeasy Kit according to the manufacturer's recommendations (Qiagen, Hilden, Germany). Gene transcription of ß-actin,* LLGL1,* and* E-cadherin* was analyzed by two-step RT-PCR: Reverse transcription was performed with 2 *μ*g of RNA (20 *μ*l total volume; Ominscript RT Kit, Qiagen) according to the recommendations of the manufacturer. One *μ*l of the cDNA was used as template for PCR-reactions. Primers applied were ß-actin-forward: 5′ - TGA CGG GGT CAC CCA CAC TGT GCC CAT CTA - 3′ and ß-actin-reverse: 5′- CTA GAA GCA TTT GCG GTG GAC GAC GGA GGG - 3′ (661 bp fragment),* LLGL1*-forward: 5′- AAG CTG TGG GCC CGC ATT GTG A- 3′ and* LLGL1*-reverse: 5′- GTC CTG GAG GAG GTC TAT GAT A - 3′ (480 bp fragment),* E-cadherin*-forward CAG GTA CAC AGC CCT AA and* E-cadherin*-reverse GCT GGC TAC AGT CAA AGT CC (641 bp). For amplification, a DNA Engine PTC200 (MJ Research, Watertown, USA) thermocycler was used. PCR cycling conditions were as follows: initial denaturation (4 min, 95°C), followed by the respective number of cycles (ß-actin: 30;* LLGL1*: 36;* E-cadherin:* 29) of denaturation (1 min, 94°C), annealing (1 min; ß-actin: 52°C;* LLGL1*: 62°C;* E-cadherin:* 57°C), and elongation (2 min, 72°C). After the last cycle, a final extension (10 min, 72°C) was added and thereafter the samples were kept at 4°C. 15 *μ*l of the products was run on a 2% agarose gel, stained by ethidium bromide and analyzed under UV light by a video densitometer.

### 2.4. Immunohistochemistry

For IHC staining of paraffin-embedded tissue sections, the avidin-biotin-complex method (LSAB+ System-HRP Kit, Dako Cytomation, Germany) was used to detect the proteins* LLGL1* (1:50; 4 hours, mouse-anti-human monoclonal antibody, Clon 5G2, Abnova, Taiwan; Polyclonal rabbit-anti-human antibody, respectively) and* E-cadherin* (1:100, 1h, Dako Cytomation, M3162). Formalin-fixed and paraffin-embedded tissues were deparaffinized and subsequently microwaved (600 W, 15 minutes) in citrate buffer (ph 6.0). After preincubation with hydrogen peroxide (LSAB+ System-HRP Kit, Dako Cytomation, Germany) and human AB plasma (Dept. of Transfusion, University of Mainz, Mainz, Germany) the primary antibodies were applied at room temperature. After incubation with the secondary antibody (LSAB+ System-HRP Kit, Dako Cytomation, Germany) the avidin-biotin complex was added and the enzyme activity was visualized with diaminobenzidine (LSAB+ System-HRP Kit, Dako Cytomation, Germany). Counterstaining was performed with haematoxylin (Roth, Karlsruhe, Germany). For negative controls of each sample, the secondary antibody was used alone. For positive controls, formalin-fixed and paraffin-embedded tissue samples of the human gastric mucosa were applied. Evaluation of the staining was performed semiquantitatively by three independent authors via light-microscopy. The intensity of staining was graded as negative: 0, weak: 1, medium: 2, and strong: 3.

### 2.5. Establishment of LLGL1-GFP Expressing Clones

We established a SNU16 cell line clone stably expressing a* GFP*-*LLGL1 *fusion protein. The SNU16 gastric carcinomatosis cell line was selected for transfection, as it has been described as carrying a deletion on chromosome 17, p11.2, the locus of* LLGL1*. Therefore, SNU16 has lost* LLGL1* expression and so was suited to investigate the effect of* LLGL1* reexpression. The* LLGL1* cDNA containing the complete open reading frame was cloned into the expression vector pcDNA3.1/NT-GFP (Invitrogen, Carlsbad, CA, USA), resulting in a* GFP-LLGL1* fusion protein. SNU16 were seeded in six-well plates and transfected with either pcDNA3.1/NT-GFP-*LLGL1* or pcDNA3.1/NT-GFP plasmid by lipofectamine 2000 reagent according to the recommendations of the manufacturer (Invitrogen, Carlsbad, CA, USA). The stably transfected SNU16-*GFP* and SNU16-*GFP*-*LLGL1* cells were selected in medium containing G418 (400 *μ*g/ml). Stable clones grew after about 4 weeks of selection and were picked and analyzed by Western blot and RT-PCR.

### 2.6. Western Blot Analysis

SNU16-*GFP* cells and SNU16-*GFP*-*LLGL1* were cultured in six-well plates. Cells were harvested, washed twice with PBS, and lysed in 1% NP-40 solution. For Western blot, 100 *μ*g of protein was loaded on a 10% SDS-PAGE gel. After separation, the gel was transferred to a PVDF membrane (Roth, Karlsruhe, Germany).* LLGL1* protein was detected with a mouse-anti-human antibody and rabbit-anti-human antibody, respectively (1:2000; overnight, 4°C; mouse-anti-human monoclonal antibody, Clon 5G2, Abnova, Taiwan; Polyclonal rabbit-anti-human antibody):* E-cadherin* was detected with a monoclonal mouse-anti-human antibody (Dako Cytomation, M3162; 1:1000; overnight, 4°C). Alpha-tubulin was analyzed with a monoclonal mouse-anti-human antibody (Sigma T5168, 1:1000; overnight, 4°C). Secondary antibodies used were goat-anti-mouse (1:10000, 1 h, room temperature; SC-2031, Santa Cruz Biotechnology, CA, USA) and goat-anti-rabbit (1:10000, 1 h, room temperature; SC-2030, Santa Cruz Biotechnology, CA, USA), respectively. For visualization, the Roti Lumin systems 1 and 2 were applied (Roth, Karlsruhe, Germany).

### 2.7. Proliferation Assays

5x10^3^ cells (SNU16-*GFP*-*LLGL1* or SNU16-*GFP*) were seeded into 96-well plates. The number of cells per well was determined daily by luminescence (Celltiter-Glo, Cell Viability assay, Promega, USA). In brief, 50 *μ*l of Cell Titer Glo were added to 100 *μ*l serum-free medium per well, followed by incubation at room temperature for 15 minutes. Luminescence was then read with a luminometer after 10 minutes. Each procedure was performed in quadruplicate.

### 2.8. Apoptosis Assay

5x10^5^ cells (SNU16-*GFP*-*LLGL1* or SNU16-*GFP*) were plated in 6-well plates. Suspension cells were collected and adherent cells trypsinized prior to fixation with 70% ethanol, staining with propidium iodide and analysis by FACS, without gating. Cells in the G1 (n) and G2/M (2n) phases of the cell cycle could be distinguished. Apoptotic cells with DNA content lower than n were quantified. Each procedure was performed in quadruplicate.

### 2.9. Adhesion Assay

For adhesion assays, SNU16-*GFP*-*LLGL1* and SNU16-*GFP* cells were used. 96-well plates had been prepared with laminin (10 *μ*g/ml, 30 minutes, room temperature, Sigma, Germany), fibronectin (40 *μ*g/ml, 30 minutes, room temperature, Sigma, Germany), or PBS and were blocked with albumin (2%, over night, 4°C, Serva, Germany), respectively. After trypsinization, 80,000 cells were seeded per 96-well and allowed to attach for 24 hours. Thereafter the medium and none-attached cells were removed. Each well was washed twice with 100 *μ*l medium. The amount of attached cells per well was determined by luminescence assay (Celltiter-Glo, Cell Viability assay, Promega, USA). Luminescence was quantified with a luminometer. Again, each procedure was performed in quadruplicate.

### 2.10. Invasion Assays

Invasion of SNU16-*GFP*-*LLGL1* versus SNU16-*GFP* cells was assayed with 24-well HTS FluoroBlok Inserts in triplet approaches (8*μ*M pore size; Becton Dickinson, USA). Membranes were covered with laminin (10 *μ*g/ml, 30 minutes, room temperature, Sigma, Germany) and blocked with albumin (2%, overnight, 4°C, Serva, Germany). In brief, 2x10^4^ cells were resuspended in serum-free DMEM and added to the upper chamber, following which DMEM with 20% FCS and 70 ng/ml SDF-1alpha was added to the lower chamber. Chambers were incubated for 24h at 37°C in a humid atmosphere of 5% CO_2_. After incubation, the number of invaded and migrated cells in the lower chamber was determined by luminescence assay (Celltiter-Glo, Cell Viability assay, Promega, USA) according to the recommendations of the manufacturer. Luminescence was quantified with a luminometer, and each procedure was performed in triplicate.

### 2.11. Subcutaneous Tumor Xenograft

Either SNU16-*GFP*-*LLGL1* or SNU16-*GFP* expressing cells (5x10^6^) were used to induce a subcutaneous tumor in 7-8 weeks old Nod-SCID mice. The mice were maintained in a laminar airflow cabinet under pathogen-free conditions. Mice were housed in microisolator cages with free access to laboratory chow and tap water. Nod-SCID mice were irradiated with 1.8 Gy one day prior to subcutaneous injection of tumor cells. Tumors grew for 6 weeks before the animals were sacrificed by carbon dioxide asphyxiation. Thereafter tumors were enucleated, embedded in paraffin, sectioned and immunostained. All animal experiments were performed in accordance with the German Animal protection Law and approved by the local responsible authorities.

### 2.12. Statistics

Patients' age was compared by calculating the mean and standard deviation of the respective subgroups. In addition, the nonparametric Wilcoxon test was applied. The *χ*^2^ test was used to compare all other patient and tumor characteristics by group. The T-test was applied to compare results obtained from functional assays. For all tests, a* P*-value of <0.05 was considered significant.

## 3. Results

### 3.1. Loss of LLGL1 Transcription in Human Gastric Cancer Cell Lines

**Figure 1:**
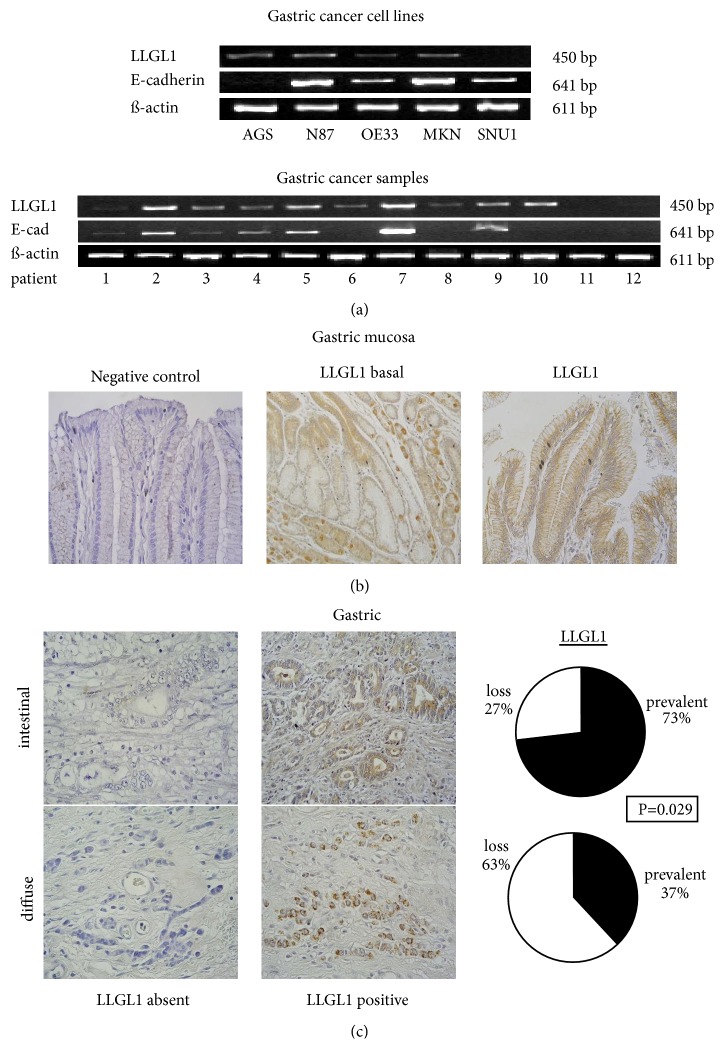
(a) Transcription profile of* LLGL1* in 5 gastric cancer cell lines and in 12 representative gastric cancer samples.* LLGL1* and* E-cadherin* reveal a similar transcription pattern in gastric cancer. (b)* LLGL1* immunostaining in gastric mucosa. Negative controls remained negative.* LLGL1* expression is absent in the basal part of the gland and strong at the foveolar top.* LLGL1* reveals a membranous localization within the mucosa cells. (c)* LLGL1* staining of gastric cancer samples. The figure depicts the expression patterns of* LLGL1* in gastric cancer (absent versus positive). Loss of* LLGL1* significantly correlated with diffuse gastric cancer.

*LLGL1* was expressed in gastric AGS, NCI-N87, OE33 and MKN cancer cell lines (Figure 1(a)). In contrast,* LLGL1* was absent in SNU16 derived from human gastric peritoneal carcinomatosis, resulting from a deletion of p11.2 on chromosome 17.

### 3.2. Tumor Characteristics and Patient Profile

The average age of all gastric cancer patients was 69 years (Table 1). 59% of all patients were male and 41% female. By histopathological grading, 23% of tumors were moderately differentiated (G1-2) compared to less differentiated (G3-G4) in 77%. The resection margins were free of residual microscopic and macroscopic tumor (R0) in 96% ofcases. According to TNM classification, half were of limited (T1/2; 52%) extent and half were locally advanced (T3/4; 48%). By pathological and clinical assessment, the majority of patients had lymphatic metastases (N1-N3; 77%). In contrast, only a minority of 27% had distant metastases (M1) at the time of surgery. The median survival was 638 days.

### 3.3. Loss of LLGL1 versus Tumor and Patient Characteristics

Loss of* LLGL1* expression occurred in 65% of gastric carcinoma samples (Table 2). TNM classification revealed a significant correlation between loss of* LLGL1* expression and distant peritoneal metastases (M1; P=0.006). In contrast, loss of* LLGL1* impacted neither on T- nor on N-status. In addition, loss of* LLGL1* showed a significant association with female gender (P=0.017) but had no relevance for the resection status (R-Status). Patients whose tumors revealed a loss of* LLGL1* showed a trend toward a shorter survival (575 days) compared to those with* LLGL1* expressing tumors (856 days; n.s.). These results revealed a significant association between loss of* LLGL1* in gastric cancer samples and distant dissemination.

### 3.4. Immunohistochemical Analysis of LLGL1 Expression in Gastric Cancer Samples

To further examine* LLGL1* expression* in vivo,* five healthy gastric mucosa samples and 39 gastric adenocarcinoma specimens (62% diffuse and 58% intestinal type according to Lauren classification) were immunostained with an* anti-LLGL1* antibody. In human gastric mucosa,* LLGL1* immunohistochemistry exhibited weak cytoplasmic and strong cortical staining along the basolateral membranes (Figure 1(b)). Interestingly,* LLGL1 *expression of gastric epithelial cells was most intense at the apical foveolar segments and absent in the basal segments of the gland.

Gastric carcinoma samples revealed varying expression intensities of* LLGL1* ranging from strong to absent (Figure 1(c)). Loss of* LLGL1* expression was significantly correlated with the diffuse type of gastric cancer (15/24; 63%) compared with the intestinal type (4/15; 27%; P=0.029). In summary, these data reveal that loss of* LLGL1 *protein staining is associated with the diffuse type gastric cancer.

### 3.5. Loss of E-Cadherin versus Tumor and Patient Characteristics

Loss of* E-cadherin* expression occurred in 68% of gastric carcinoma samples (Table 3). TNM classification showed a trend between loss of* E-cadherin* expression and distant metastases (P=0.07). In contrast, loss of* E-cadherin* impacted on neither T- nor N-status. However, loss of* E-cadherin* revealed a significant association with female gender (P=0.0017) but had no relevance for the resection status (R-Status). Patients whose tumors revealed loss of* E-cadherin* showed a trend to reduced survival (614 days) compared to those with* E-cadherin* expression (798 days; n.s.). These results underline the relevance of* E-cadherin* for gastric cancer dissemination.

### 3.6. Correlation between Loss of LLGL1 and E-Cadherin Expression

Loss of* LLGL1 *significantly correlated with loss of* E-cadherin* expression. Similarly, loss of* E-cadherin* expression revealed a significant correlation with loss of* LLGL1* expression (P=0.00009, respectively; Table 4). These results implicate a common regulation of the adhesion molecules* LLGL1* and* E-cadherin*.

### 3.7. Functional Analysis Using LLGL1-GFP Stably Expressing SNU16 Cell Line

**Figure 2:**
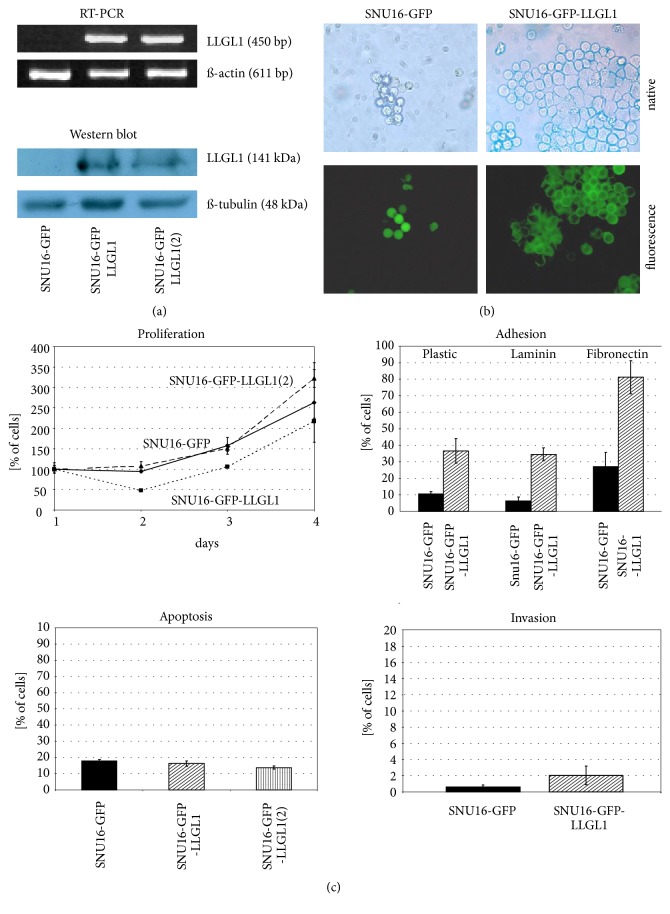
(a) RT-PCR and Western Blot confirms successful transfection of SNU16 cancer cells with* LLGL1-GFP*. (b) Fluorescence microscopy confirms the membranous accumulation of* GFP*-*LLGL1* fusion protein in comparison to the cytoplasmic* GFP* localization of* GFP* only expressing cells. (c)* LLGL1* reexpression in SNU16 did not impact on proliferation, apoptosis, or invasion. However, reexpression of* LLGL1* in SNU16 resulted in a significant increase of adhesion to plastic and extracellular matrix proteins laminin and fibronectin.

RT-PCR and Western blot analysis of stably transfected SNU16 cells confirmed the expression of the* LLGL1-GFP* protein with the calculated molecular mass in contrast to* GFP* only expressing clones (Figure 2(a)). Two different* LLGL1-GFP *expressing clones were selected, SNU16-GFP-LLGL1 and SNU16-GFP-LLGL1(2).

Expression of* LLGL1* did not modify the transcription or the protein expression level of* E-cadherin*, implicating that both proteins are independent downstream targets of a common regulator. SNU16 cells expressing* GFP-LLGL1* revealed an intense submembranous accumulation of* GFP*-*LLGL1* indicating a cortical localization of* LLGL1*, which was enhanced in regions of cell-cell contact (Figure 2(b)). In contrast SNU16-*GFP* cells depicted a cytoplasmic localization of* GFP* (Figure 2(b)).

Functional analyses did not depict any significant impact of* LLGL1* on proliferation (Figure 2(c)). Luminescence analyses after 3 days of cell culture revealed the following results: SNU16-GFP: 263% (+/- 97%), SNU16-GFP-LLGL1: 218% (+/- 7%; P=0.53; n.s.), and SNU16-GFP-LLGL1(2) 322% (+/- 22%; P=0.4; n.s.).

Similarly, analyses of apoptosis did not reveal any significant impact of* LLGL1* expression (Figure 2(c)): SNU16-*GFP*: 17,8% (+/- 0,98%), SNU16-GFP-LLGL1: 16,28% (+/- 1,69%; n.s.), and SNU16-GFP-LLGL1(2) 13,81% (+/- 1,93%; n.s.).

Interestingly, expression of* LLGL1* significantly enhanced the adhesion of cancer cells to plastic, laminin, and fibronectin (Figure 2(c)). Adhesion analyses revealed following results: for plastic surface: SNU16-GFP-LLGL1: 37% (+/- 7%). SNU16-*GFP*: 11% (+/- 1%; P=0.044); for laminin coating: SNU16-GFP-LLGL1: 35% (+/- 4%), SNU16-*GFP*: 6% (+/- 2%; P=0.028); and for fibronectin coating: SNU16-GFP-LLGL1: 81% (+/- 10%) versus SNU16-*GFP*: 27% (+/- 9%; P=0.0025).

However,* LLGL1* expression did not impact significantly on invasion, as measured by invasion analyses (Figure 2(c)): SNU16-*GFP*: 0,6% (+/- 0,2%) versus SNU16-GFP-LLGL1: 2,1% (+/- 1,2%; P=0.13; n.s.). The slight increase can be considered to be a result of increased adhesion rather than of augmented invasion.

In summary, these functional assays demonstrate that* LLGL1* expression has no impact on cell proliferation, apoptosis, or invasion but does significantly increase cell adhesion. These observations are in accordance with the hypothesis that loss of* LLGL1* expression contributes to cancer dissemination and progression by loss of cell-to-cell junction mediating adherence

### 3.8. Subcutaneous Tumor Growth of SNU16^LLGL1-GFP^ Cells Stably Expressing SNU16 Cell Line in a Xenograft Model

**Figure 3:**
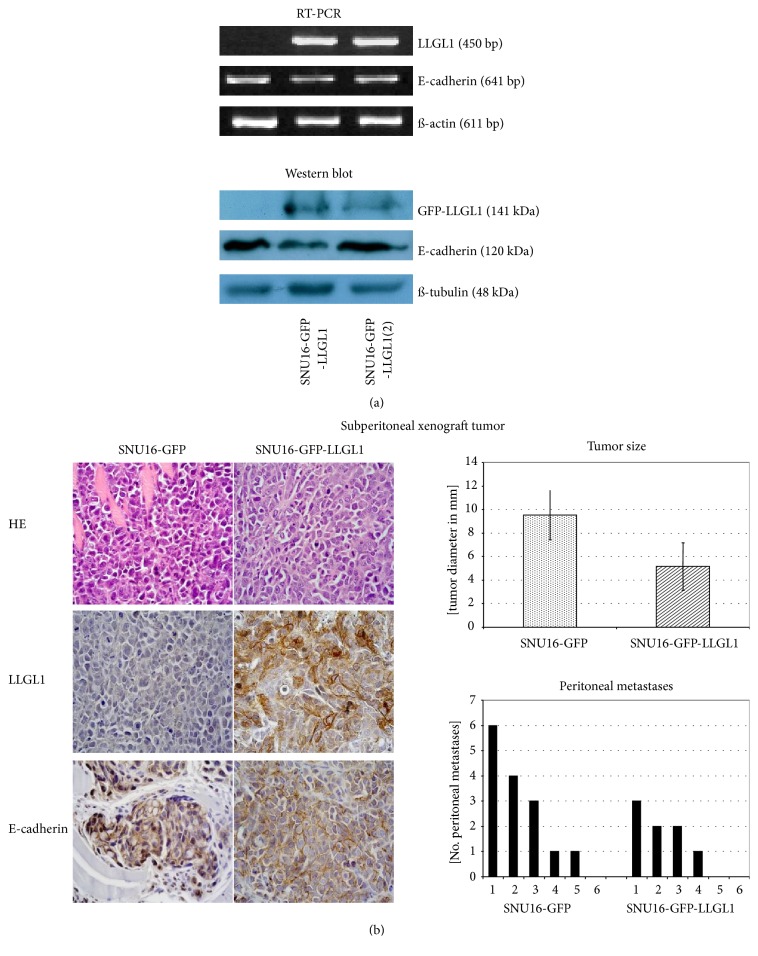
(a)* LLGL1* expression did not impact on expression of* E-cadherin*. (b) Subcutaneous xenograft tumor. Immunohistochemistry revealed a predominantly membranous staining of* LLGL1* in* GFP-LLGL1* expressing tumors, in contrast to* GFP only* expressing tumors. Expression of* LLGL1* did not alter the expression intensity of* E-cadherin,* but increased membranous redistribution of* E-cadherin*. Expression of* LLGL1* did not alter proliferation or grading in vivo.

SNU16-GFP-LLGL1 and SNU16-*GFP* expressing cells were used to induce subcutaneous tumors in Nod-SCID mice (Figure 3(b)). Immunohistochemistry revealed a predominantly membranous staining of* LLGL1* in* GFP-LLGL1* expressing tumors, in contrast to* GFP only* expressing tumors. Expression of* LLGL1* did not alter the expression intensity of* E-cadherin,* but increased membranous redistribution of* E-cadherin*. However,* LLGL1* impacted on neither tumor size (*LLGL1-GFP versus GFP*; 11mm versus 10mm) nor differentiation of the tumor, indicated by tumor grading (G3, respectively). These data confirm that* LLGL1* does not impact on proliferation or on epithelial-mesenchymal transition (EMT), but increases adhesion as depicted in our functional analyses.

## 4. Discussion

We initiated this study to investigate the relevance of* LLGL1* expression for gastric cancer development and progression. Specifically, we were interested to know whether* LLGL1* expression is lost in gastric cancer and if so whether loss of* LLGL1* expression occurs in a larger context of cellular deadhesion. Therefore, we analyzed the expression and regulation of* E-cadherin* in parallel.

We have previously described the loss of* LLGL1* expression in a large cohort of colorectal cancer patients and its impact on tumor cell dissemination* in vivo* and* in vitro* [30]. Matching our current observations in gastric cancer,* LLGL1* expression did not impact on proliferation, cell cycle, or apoptosis in colorectal cancer. Further studies revealed that loss of* LLGL1* expression is lost in various cancers [24, 30, 31]. In addition, Tsuruga and colleagues described loss of* LLGL1* expression in endometrial cancer and reported a correlation with metastatic disease [32]. Furthermore, loss of* LLGL1* expression is correlated with reduced overall survival in pancreatic and squamous lung cancers [34, 36].

Our current data are supported by these reports, and prove an interesting link between* LLGL1* and gastric cancer, underlining the relevance of cellular deadhesion in the context of tumor cell dissemination for the following reasons:

1. We found that* LLGL1* transcription was lost in 65% of all gastric cancers and that its loss correlated significantly with distant dissemination, particularly with peritoneal carcinomatosis in patients.
2. Loss of* LLGL1* expression significantly correlated with the diffuse type of gastric cancer as compared to the better differentiated intestinal type according to the Lauren classification. These results match the findings of the second human Drosophila homologue,* LLGL2*, as was recently reported [41].
3. We found a highly significant correlation between loss of* LLGL1* and loss of* E-cadherin* expression, respectively. Loss of* E-cadherin* expression had previously been correlated with the diffuse type gastric cancer in a landmark paper by Becker and colleagues back in 1994 [13]. Since then, multiple groups described this clinical association and linked the loss of* E-cadherin* expression with familial diffuse gastric cancer [15]. Downregulation or loss of* E-cadherin* decreases the strength of cellular adhesion within a tissue and induces activation of the* ß-catenin* pathway, resulting in increased cellular motility and invasion [16]. A similar association was found for* LLGL2* in other studies [41].
4. *LLGL1* staining revealed epithelial staining in healthy gastric mucosa, which was strongest at the foveolar top and weakest at the bottom of crypts. Hence,* LLGL1* expression is likely induced during maturation and differentiation of epithelial cells. These data resemble the observations which we made in colonic mucosa [30]
5. *In vitro* expression of* LLGL1* protein resulted in a significant increase of cellular adhesion while not it had no impact on proliferation, apoptosis or invasion in gastric carcinoma. These results are in contrast to the findings of Song et al. who showed a reduced proliferation and increased apoptosis in* LLGL1* reexpressing esophageal cancer cells [37]). Beside the localization, cell origin (squamous versus adenocarcinoma) and architecture (mono- versus multilayer epithelium) are the main differences, which could result in these findings. Thus, loss of* LLGL1* might contribute to the mechanical dissemination of cancer cells as seen in diffuse gastric cancer with consecutive peritoneal carcinomatosis.
6. Our xenograft tumors revealed no impact of* LLGL1* on grading, but did reveal an increased membranous accumulation of* E-cadherin*. Both* GFP* and* LLGL1-GFP* expressing tumors depicted a dedifferentiated phenotype, graded as G3. Thus,* LLGL1* does not control either differentiation or EMT.
7. SNU16 cells obtained from malignant ascites grow as suspension cells and reveal a loss of* LLGL1* expression while maintaining expression of* E-cadherin*. Loss of* LLGL1* is due to a deletion on chromosome 17 (p11.2; ATCC, USA). Reexpression of* LLGL1* enabled these cells to grow in clusters with an epithelial phenotype, reflecting increased cellular adhesion. These findings are in accordance with descriptions in mammary epithelial cells. Knockdown of* LLGL1* expression was correlated with mesenchymal phenotype and reduced acinar formation [42]. Therefore, a role of* LLGL1* in reinforcement of epithelial junctions or desmosomes should be postulated, demanding further analyses [17, 43].

Our results point toward recent mechanistic findings from Drosophila's* LLGL1* homologue* l(2)gl*. It has been shown that basolateral* l(2)gl* is part of the cortical membrane cytoskeleton stabilizing epithelial structures. Here, l*(2)gl* forms a complex with* Dlg* and* cribble* crucial to the formation of epithelial junctions such as tight junctions in mammalian epithelial cells [17]. In contrast, apical* l(2)gl *plays a critical role in induction of migration [25, 27] Among the strongest inductors of chemotaxis-mediated migration are chemokine receptors and their ligands, such as* CXCR4* and* CXCL12* [44, 45]. Activation of diverse chemokine receptors results in activation of the* PI3K* pathway which again results in activation of* aPKC* and phosphorylation of apical* l(2)gl *[44, 45]. Phosphorylated* l(2)gl* dissociates from the apical cytoskeleton in order to become a member of the polarity complex (*L(2)gl*,* Par6,* and* aPKC) *[25–27]. For cell migration, the polarity complex concentrates integrin clusters in the anterior aspect of the cell, resulting in polarized adhesion and transmigration.

In summary, the development of gastric cancer is associated with progressive loss of epithelial structure, cell polarity, and decreased cell-to-cell contact. The available information on* LLGL* proteins from studies in Drosophila and humans supports the theory that* LLGL1* contributes to maintenance of epithelial integrity. The coregulation with* E-cadherin* implicates a relevant role for* LLGL1* in epithelial junctions or desmosomes. Taken together with the results presented in this paper, a role for* LLGL1 *in diverse human malignancies is predicted, thus warranting further investigations.

## Figures and Tables

**Table 1:** Patient and tumor characteristics.

	Patient characteristics
*Total number*	*56*

*Median age (years)*	69

*Gender*	
Female	23(41%)
Male	33(59%)

*T – Status*	
1	3(5%)
2	26(47%)
3	23(41%)
4	4(7%)

*N – Status*	
0	13(23%)
1	16(29%)
2	13(23%)
3	14(25%)

*M – Status*	
0	41 (73%)
1	15 (27%)

*R-Status*	
0	54 (96%)
1	2 (4%)

*Median survival (days)*	638

**Table 2:** Patient and tumor characteristics dependent on intensity of *LLGL1* expression.

	*LLGL1* expression		*statistics*
	Absent	Present	
*Total number*	36(64%)	20(36%)	

*median age (years)*	68	70	*n. s. *

*Gender*			
Female	19(53%)	4(20%)	*P=0.017*
Male	17(47%)	16(80%)	

*T – Status*			
1+2	19(53%)	10(50%)	*n. s. *
3+4	17(47%)	10(50%)	

*N – Status*			
0	6(20%)	7(35%)	*P=0.11; n. s.*
+	30(80%)	13(65%)	

*M – Status*			
0	22(61%)	19(95%)	*P=0.006*
1	14(39%)	1(5%)	

*R-Status*			
0	34(94%)	52(100%)	*n. s.*
1	2(6%)	0(0%)	

*Median Survival (days)*	575	856	*P=0.36; n. s.*

**Table 3:** Patient and tumor characteristics dependent on intensity of *E-cadherin* expression.

	*E-cadherin* expression		*statistics*
	Absent	Present	
*Total number*	38(68%)	18(32%)	

*median age (years)*	67	73	*n. s. *

*Gender*			
Female	21(55%)	2(11%)	*P=0.00171*
Male	17(45%)	16(89%)	

*T – Status*			
1+2	17(45%)	12(67%)	*n. s. *
3+4	21(55%)	6(33%)	

*N – Status*			
0	17(45%)	12(67%)	*P=0.125, n. s.*
+	21(55%)	6(33%)	

*M – Status*			
0	25(66%)	16(89%)	*P=0.07*
1	13(34%)	2(11%)	

*R-Status*			
0	36(95%)	18(100%)	*n. s.*
1	2(5%)	0(0%)	

*Median Survival (days)*	614	798	*n. s.*

**Table 4:** *LLGL1* expression versus *E-cadherin* expression.

	*LLGL1* expression		*statistics*
	Absent	Present	
*E-cadherin* expression			
absent	31	7	
present	5	13	*P=0.00009 *

## Data Availability

All experimental data used to support the findings of this study are included within the article

## Abbreviations

bp: Base pair
CRC: Colorectal carcinoma
*LLGL1*: Lethal(2) giant larvae protein homologue 1
*l(2)gl*: Lethal giant larvae
PCR: Polymerase chain reaction
RT: Reverse transcription
IHC: Immunohistochemistry
FACS: Fluorescence-activated cell scanning
FCS: Fetal calf serum
DMEM: Dulbecco's Modified Eagle's medium
SDS-PAGE: Dodecyl sulfate polyacrylamide gel electrophoresis
PVDF: Polyvinylidene difluoride
DNA: Deoxyribonucleic acid
RNA: Ribonucleic acid
